# The Heat Stability of Hepatitis B Virus: A Chronological Review From Human Volunteers and Chimpanzees to Cell Culture Model Systems

**DOI:** 10.3389/fcimb.2020.00032

**Published:** 2020-02-04

**Authors:** Jochen Steinmann, Joerg Steinmann, Eike Steinmann

**Affiliations:** ^1^Brill + Partner GmbH Institute for Hygiene and Microbiology, Bremen, Germany; ^2^Institute of Medical Microbiology, University Hospital of Essen, Essen, Germany; ^3^Institute of Clinical Hygiene, Medical Microbiology and Infectiology, General Hospital Nürnberg, Paracelsus Medical University, Nuremberg, Germany; ^4^Department for Molecular and Medical Virology, Faculty of Medicine, Ruhr University Bochum, Bochum, Germany

**Keywords:** hepatitis B virus, thermostability, inactivation, cell culture, moist heat, A0 value

## Abstract

During the World War II jaundice and hepatitis in the US army were observed after vaccination with the yellow fever vaccine containing human plasma for stabilization. This led to first heat experiments with volunteers without knowledge of the causative agents. Finally, experiments of human serum with volunteers and chimpanzees led to the conclusion that the hepatitis B virus (HBV) which had been identified as the responsible agent of the contamination of the vaccine, could not be inactivated at 98°C after 1 min, whereas 2 min in two chimpanzees were enough. Meanwhile, a cell culture system became available showing that 2 min exposure time is not enough depending on the virus strain used whereas 5 min means complete inactivation of HBV. The great stability of the blood-borne HBV was also of interest in hospital hygiene due to the use of moist heat for disinfection of heat-stable medical devices in washer-disinfectants. The requirements for washer-disinfectors and the parameters describing disinfection with moist heat are defined in the EN ISO 15883. In this standard, the efficacy of this thermal disinfection is described by the A_0_ value. For heat-resistant viruses a higher A_0_ = 3,000 is often recommended including semi-critical instruments that undergo thermal disinfection and no final sterilization. All experiments including volunteers, chimpanzees and now cell culture were performed with greater A_0_ values than 3,000. Therefore, an A_0_ value of 3,000 e.g., being reached by 90°C and 5 min in washer-disinfectants, can easily elevated to 6,000 by prolongation of the exposure time to 10 min. In contrast to the different laboratory experiments with high virus titers it should be considered that in practice the necessary cleaning step upfront will help to reduce virus load and then protect the personnel in the medical area.

## HBV and Thermal Disinfection

Despite the availability of vaccination, infections with hepatitis B virus (HBV) are still a severe global health burden, with ~2 billion infected individuals and more than 250 million carriers worldwide. HBV, a small, enveloped, hepatotropic DNA virus is the major cause of chronic liver diseases, the primary causative agent of hepatocellular carcinoma (HCC) and responsible for 887,000 deaths worldwide annually (WHO, 2017). Chronic hepatitis B (CHB) is still an incurable disease.

HBV is highly contagious circulating in patients' blood with a minimal infectious dose of only 10 genomes (Komiya et al., 2008). Healthcare workers are at constant risk of acquiring HBV infection from occupational exposure. Moreover, nosocomial transmissions of HBV with an increasing number of outbreaks have been reported worldwide over the past few years. Common routes of HBV transmission include the use of multi-dose vials (Fisker et al., 2006; Sauerbrei, 2014), dental or biopsy equipment (Drescher et al., 1994), dialysis unit (Carrilho et al., 2004), contaminated finger-stick devices (Bender et al., 2012; Lanini et al., 2012), acupuncture needles (Walsh et al., 1999), reuse of syringes (Thompson et al., 2009), endoscopes (Santos et al., 2004) and unsafe surgical and injection procedures (Welch et al., 1989; Goldmann, 2002; Buster et al., 2003; Redd et al., 2007). Most of these HBV infections remain clinically asymptomatic and are not immediately recognized by medical personnel.

HBV can survive and remain infectious on environmental surfaces for at least 7 days (Bond et al., 1981). Since moist heat is known to kill microorganisms including viruses, thermal disinfection in the hospital for decontamination of heat-stable reusable medical devices is an essential process in the prevention of nosocomial infections including HBV. After a cleaning step, the thermal disinfection of medical devices is running in automated washer-disinfectors machines.

The requirements for washer-disinfectors and the parameters describing disinfection with moist heat are defined in the European (EN) and International Organization for Standardization (ISO) 15883 (*DIN EN ISO 15883-2:2009-09*)^1^. In this standard, the efficacy of this thermal disinfection is described by the A_0_ value. The A_0_ value is defined as Σ10^(T−80)/z)^ × Δt. This value is based on exposure times and temperatures necessary for inactivation of microorganisms with a defined z value. By this, various time-temperature relationships can be compared. A low value of A_0_ = 60 is regarded as minimum for devices coming into contact with intact skin and pathogens like heat-sensitive viruses, whereas A_0_ = 600 is necessary for surgical instruments (*DIN EN ISO 15883-2:2009-09*)^1^. For heat-resistant viruses a higher A_0_ = 3,000 is often recommended including semi-critical instruments that undergo thermal disinfection and no final sterilization (Röhm-Rodowald et al., 2013). In the past, the conclusion that a high A_0_ value = 3,000 must be chosen if an efficacy against heat-resistant viruses such as HBV is necessary was questioned (Rosenberg, 2003). Furthermore, in the discussion of the necessary A_0_ value for heat resistance viruses it also has to be considered that the amount of HBV in the human serum of chronic carriers can be very high and is not part of the A_0_ value. Virus titers up to 10^9^/mL HBV DNA copies can be found in sera of patients. However, disinfection is always performed after a cleaning step which should reduce the number of microorganisms on the instruments by some orders of magnitude. This reduction has important consequence for the requirements of thermal heating. Moreover, this is a very important step for the protection of processing personnel (Rosenberg, 2003). However, before the concept of the DIN EN ISO 15883 was established the choice of biological indicators was the basis of describing general requirements regarding disinfection with moist heat in washer-disinfectors. The high thermal resistance of HBV as a blood-borne virus was already a key question when the A_0_ concept was introduced first.

## A Historical Overview of the Thermostability of HBV

A recent study described the heat stability of HBV resulting from cell culture assays under defined laboratory conditions (König et al., 2019). The reported data on heat-resistant HBV were the first using patient-derived viruses and a HBV cell culture model system in parallel. These results are important for the requirements of thermal disinfection in washer-disinfectors using moist heat. All other data which are already used for recommendations of thermal disinfection in the past were based on experiments with human volunteers and chimpanzees due to the lack of a suitable cell culture system. Therefore, the comprehensive story of heat stability of HBV can be continued and perhaps finished.

**Figure 1:**
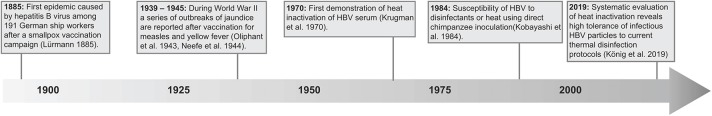
Timeline of landmarks HBV transmission and inactivation studies.

Our first knowledge of HBV heat stability started with several experiments with human volunteers after jaundice and hepatitis occurring after yellow fever vaccination in World War II. A vaccine might contain viruses if blood-products like plasma were used for production. Already in 1885, Lürmann described vaccination against smallpox glycerinised with human lymph in a shipyard in Bremen resulting in 191 men becoming jaundiced within 2–8 months (Lürmann, 1885). This was one of the first observations in the nineteenth century without knowledge of the etiologic agent that blood containing products like vaccines were responsible for epidemic transmission of homologous serum hepatitis (Figure 1). Another large outbreak was then described by the office of the Surgeon General of the US army in April 1942 (Office of the Surgeon General, 1942). At that time cases of jaundice and hepatitis occurred in US military personnel who received yellow fever vaccine during World War II. The observed symptoms were linked to a specific lot of vaccine named 17D (Oliphant et al., 1943; Neefe et al., 1944a). An experiment with nine volunteers for confirmation and detailed clinical observations followed (Neefe et al., 1944b). Finally, 23,664 cases of hepatitis in Armed Forces personnel were reported in 1944 (Sawyer et al., 1944). In parallel, identical observations after vaccination were made in Brazil (Fox et al., 1942). At that time human immune serum was used to stabilize the vaccine after being heating at 56°C for 30–60 min. Consequently, the use of human serum in vaccine production was stopped. In a review describing mortality and morbidity among military personnel between 1930 and the end of World War II after yellow fever vaccination in total 49,233 cases of jaundice or hepatitis were reported among US troops (Thomas et al., 2013). Consequently, studies with volunteers were initiated to examine the effect of heat to the unknown agent of homologous serum hepatitis. Sawyer et al. (1944) and MacCallum and Bauer (1944) confirmed that heating at 56°C for 30–60 min was not enough for complete inactivation of the responsible agent. At that time the agent was described as filterable and resistant to heating to 56°C for 30 min (Havens, 1945). In contrast, the virus of the homologous serum hepatitis appeared to be inactivated in a human albumin solution at 60 and 64°C for 10 h (Gellis et al., 1948). Later, this activity by heating against HBV was not confirmed by Shikata et al. showing that heat treatment at 60°C for 10 h provided only a four log_10_ reduction of virus titer, but no complete inactivation when inoculated in chimpanzees (Shikata et al., 1978). In addition, introducing a shortage of incubation time at 60°C to 4 h samples of plasma containing the agent of homologous serum hepatitis also retained the ability to produce hepatitis in volunteers (Murray and Diefenbach, 1953). Finally, the studies of Krugman et al. at the Willowbrook State School for mentally disabled children in 1970 when describing two types of hepatitis MS-1 and MS-2, HBV serum underwent heat inactivation at 98°C for 1 min after 43 s of heat-up time in a flask over an electric burner (Figure 1). After cooling to room temperature in 25 min, serum was inoculated (0.1 mL) to each of 29 volunteers (Krugman et al., 1970). The titer was given as 10^7.5^ chimpanzee infective dose (CID)_50_ mL before 1:10 dilution and inoculation of 0.1 mL. Later, the A_0_ value for this experiment was calculated to a value of 3,786 (Uetera et al., 2010). The data showed no evidence of infection clinically or in the laboratory tests available in this year. But in 1979, a more sensitive test (radioimmunoassay) revealed three sub-clinically infected volunteers without liver involvement. At that time the frozen sera were re-examined for HBsAg (Krugman et al., 1979).

In another experiment serum with subtype adr of HBV after 1:1,000 dilutions was treated at 98°C for 2 min after 4 min of heat-up time in a thermostat bath. After cooling in an ice-water bath the sample was inoculated two chimpanzees without any evidence of infection (Kobayashi et al., 1984). The calculated A_0_ value for this moist heat treatment of 10^5^ CIC_50_/mL was 7,571 following the calculation of Uetera et al. (2010).

To confirm or disprove the data of Krugman et al. and Kobayashi et al. it was recently shown with a newly developed HBV cell culture system that cell culture-derived virus was still detectable after 2 min incubation at 98°C (Figure 1). The tests were run in thin-wall PCR tubes in a thermal cycler with immediate cooling to ice (König et al., 2019). Inactivation profiles of three HBV isolates from patients showed that one isolate was inactivated after 1 min, whereas the isolates from the two other patients could not be inactivated after 2 min exposure time. After treatment at 98°C for 5 min no virus infection was detectable testing all isolates (König et al., 2019).

The difference between the data from human volunteers, chimpanzees, and cell culture might partly be derived from the technical standard used in the laboratories at different times. In 1970, the heating was performed with an electric burner with 43 s of heat-up time and cooling down time within 25 min before inoculation of the volunteers (Krugman et al., 1970). In 1984, the heating was performed in a bath of liquid paraffin after 4 min heat-up time and a rapidly cooling in an ice-water bath before chimpanzees were inoculated (Kobayashi et al., 1984). In the recent study in 2019 with cell culture, thermo heaters were used which allowed a more precise control of all parameters used compared to those in the past (König et al., 2019).

Furthermore, it cannot be excluded, that different virus strains/genotypes influence thermal stability, since the different studies used various virus strains. Krugman et al. used the MS-2 strain, while Kobayashi et al. performed the experiments with the JTB001 strain (subtypes adr) in 1984. In the recent study, the different virus isolates from the patient material were genotype C, whereas the other viruses derived from HepAD38 cells were genotype D.

Besides the technical changes described and the different viruses incorporated, the HBV titers will influence the results in the largest extent. In the voluntary active immunization of volunteers, a 1:10 dilution of an infectivity titer of 10^7.5^ was used (Krugman et al., 1970). Both chimpanzees received a 1:1,000 dilutions of a serum with 10^8^ units/mL (Kobayashi et al., 1984). In the recently published study of Koenig et al. virus titers ranged from 5.8 × 10^10^ GEq/mL (genome equivalent) for the cell culture virus to 1.1 × 10^7^ to 5.5 × 10^9^ GEq/mL for patient-derived viruses.

Uetera et al. calculated that the A_0_ value was 3,786 when the experiments with the volunteers at the Willowbrook State School were performed (Uetera et al., 2010). Here no complete inactivation was observed after 1 min exposure time. In the chimpanzee infection model with 2 min exposure the A_0_ reached 7,571 thus confirming virus inactivation. Concerning the failure in the study at the Willowbrook State School in comparison to the chimpanzee model, it was argued that the serum infectivity titer of the human volunteer study was more than 30 times higher than that used in the animal model (Uetera et al., 2010). In the study of Koenig et al. an A_0_ value resulted of 5,400 without complete abrogation of infectivity within 2 min exposure time using high titers for the cell culture and the patient-derived viruses.

Nowadays, the capacity of washer-disinfectors must be not less than A_0_ = 3,000 and at least A_0_ = 600 (*DIN EN ISO 15883-2:2009-09*)^1^. In summary, all experiments reviewed here with volunteers, chimpanzees and cell culture models now confirm a necessary A_0_ value of >3,000. An A_0_ value of 3,000 in washer-disinfectors is often reached by 90°C and 5 min exposure time. After prolongation to 10 min an A_0_ value of 6,000 can be reached. Furthermore, the material compatibility must additionally be considered and the important fact that a cleaning step up front is included which also will increase the safety of the staff thus lowering a possible viral contamination with HBV (Röhm-Rodowald et al., 2013).

## Conclusion

In conclusion, there are data available derived from volunteers and chimpanzees concerning the heat stability of HBV in serum under not ideally defined conditions. A newly developed cell culture system should allow more precise information on this characteristic of an important virus in human medicine. The pronounced heat stability after experiments with volunteers and chimpanzees in the past was even surpassed. Finally, the still on-going discussion on thermal stability of HBV with consequences for moist heat disinfection can be finished.

## Author Contributions

ES, JocS, and JoeS drafted the article.

### Conflict of Interest

JocS was employed by Brill + Partner GmbH. The remaining authors declare that the research was conducted in the absence of any commercial or financial relationships that could be construed as a potential conflict of interest.

## Footnotes

^1^DIN EN ISO 15883-2:2009-09: Washer-disinfectors - Part 2: Requirements and tests for washer-disinfectors employing thermal disinfection for surgical instruments, anaesthetic equipment, bowls, dishes, receivers, utensils, glassware, etc. DIN 15883-2 (2009).
